# THE RAISE FAMILY CAREGIVER ADVISORY COUNCIL: STRATEGIES TO BOLSTER CAREGIVERS’ FINANCIAL SECURITY

**DOI:** 10.1093/geroni/igac059.1130

**Published:** 2022-12-20

**Authors:** Pamela Nadash

## Abstract

The RAISE Family Caregiving Advisory Council, created under the Recognize, Assist, Include, Support, and Engage (RAISE) Family Caregivers Act (2018) has been tasked to support the Secretary of Health and Human Services in developing a national family caregiving strategy. The Council began by (in 2021) identifying five key Goals critical to supporting family caregivers, which were reported to Congress in the Council’s Initial Report; the next step (in 2022) was to identify how these Goals are to be operationalized via specific actions, as well as the stakeholders that needed to be involved. This symposium discusses Goal 4, which states that “Family caregivers’ lifetime financial and employment security is protected and enhanced,” a goal incorporating diverse components, including federal legislation (expanding FMLA, for example), enhancing workplace security for working caregivers, and ways to pay family caregivers for providing supportive services. The first paper, by Salom Teshale, PhD, will provide an overview of the Council’s work and the strategies that have been chosen to support the overall national strategy. The second paper, by Eileen J. Tell, MPH, will describe strategies to improve the ability of caregivers to remain and thrive in the workplace. Pamela Nadash, PhD, will report on the research that identified the expansion of self-directed programs to incorporate payment for family caregivers as key, and the fourth paper by Rani Snyder will conclude by identifying the research needed to move these efforts forward. Greg Link of the Administration for Community Living will act as discussant.

